# Prevalence and determinants of breast self-examination practices among women in their reproductive age in Namibia: an analysis of the 2013 Namibia demographic and Health Survey

**DOI:** 10.1186/s12889-023-14985-5

**Published:** 2023-01-05

**Authors:** Joshua Okyere, Nutifafa Eugene Yaw Dey, Kenneth Owusu Ansah, Sonu Elijah Thywill, Precious Adade Duodu

**Affiliations:** 1 Department of Population and Health, University of Cape Coast, Cape Coast, Ghana; 2 Department of Nursing, College of Health Sciences, Kwame Nkrumah University of Science and Technology, Kumasi, Ghana; 3 Department of Psychology, University of Ghana, P.O. Box LG 84, Legon, Ghana; 4 Department of Psychology, Arizona State University, Tempe, AZ USA; 5 Department of Psychology, Stellenbosch University, Stellenbosch, South Africa; 6 Department of Microbiology and Immunology, School of Medical Sciences, University of Cape Coast, Cape Coast, Ghana; 7 Department of Nursing and Midwifery, School of Human and Health Sciences, University of Huddersfield, Queensgate, Huddersfield, England, UK

**Keywords:** Breast cancer, Self-examination, Demographic and health survey, Women

## Abstract

**Background:**

In resource-constrained settings like Namibia, breast self-examination (BSE) is considered an important cost-effective intervention that is critical to the early detection of breast cancer, and better prognosis. Even though BSE is a simple, quick, and cost-free procedure, its practice varies across different contexts. Knowing the determinants of BSE is necessary to inform the implementation of policies and targeted interventions to improve the practice across the population. In Namibia, estimating the magnitude of BSE practice and its determinants using nationally representative data has received limited scholarly attention. Hence, the present study sought to examine the prevalence and determinants of BSE practices among women of reproductive age in Namibia.

**Methods:**

This study relied on the 2013 Namibia Demographic and Health Survey (NDHS), analysing data from women aged 15–49 years. Statistical analyses including bivariate and multivariate logistic regression analyses were done using Stata version 14. Adjusted odds ratio (AOR) and confidence interval (CI) are presented. We followed the ‘Strengthening the Reporting of Observational Studies in Epidemiology’ (STROBE) statement.

**Results:**

Only 30.67% of the respondents practiced BSE. The odds of performing BSE were higher among those with health insurance coverage [AOR = 1.59, 95% CI: 1.34, 1.89], those who were separated from their spouses [AOR = 1.36, 95% CI: 1.03, 1.80], those within the richest wealth index [AOR = 1.69, 95% CI: 1.23, 2.33, *p* ≤ 0.001], and among Catholics [AOR = 0.84, 95% CI: 0.71, 0.98]. Women with secondary [AOR = 2.44, 95% CI:1.78, 3.35, *p* ≤ 0.001] or higher education [AOR = 3.39, 95%CI:2.24, 5.14] had higher odds of performing BSE. Women aged between 20–49 years had a significantly higher likelihood to practice BSE. Compared to women who live in Khomas, those living in Erongo, Karas, and Omaheke, were more likely to practice BSE than those in Kavango, Ohangwena, Omusati, Oshana, and Oshikoto.

**Conclusion:**

We conclude that the determinants of BSE practice are age, educational level, marital status, health insurance coverage, religion, mobility in the last 12 months, early sexual debut, parity, household wealth index, and region of residence. Any policy or intervention to improve BSE practice among Namibian women of reproductive age must target adolescent girls, those with no formal education, those without health insurance coverage, multiparous women, and those in the poorest wealth index.

## Background

Cancer is a global public health concern that accounts for one in six deaths worldwide [1]. Among all the types of cancers, breast cancer is regarded as the second commonest form of cancer [2]. Estimates on the global incidence and survival of breast cancer report that in 2018 alone, nearly 2 million new cases were reported worldwide [3]. Cancer of the breast is also reported to be the most common malignancy in low-and-middle-income countries (LMICs); often accompanied by a disproportionately high burden of breast cancer-related mortalities [4]. Evidence shows that 70 percent of breast cancer cases across the globe are reported in LMICs [5]. The situation in Namibia is not too different from the global trends and patterns of exacerbation in the incidence and mortality due to breast cancer. For instance, evidence from the Namibian National Cancer Registry (NNCR) shows that breast cancer is the leading cancer among women with an average of 549 new cases each year [6].

Several factors have been documented to be significantly associated with the risk of developing breast cancer. These factors include having a sedentary lifestyle, low intake of fruits and vegetables, tobacco use, alcohol consumption and red meat consumption, and non-modifiable factors including genetics, age, parity and race [7]. This implies that by altering modifiable factors toward healthy lifestyles, women can reduce their risk of developing breast cancer. However, this does not take away the risks posed by non-modifiable factors. Therefore, adopting secondary preventive measures like cancer screening and human papillomavirus (HPV) vaccination provides extra protection while facilitating the early detection and treatment of malignancy [8].

Basically, there are two forms of breast cancer screening: screening through examination by a doctor or health professional, and breast self-examination (BSE) [9, 10]. Screening through examination by a health professional is the recommended gold standard; it includes methods such as mammograms, clinical breast examination, ultrasounds and magnetic resonance imaging (MRI) [11]. However, the implementation of examination by a health professional has been problematic in resource-constrained countries [11]. Therefore, having BSE to complement the screening process is a much more viable option. BSE is a low-cost and non-invasive method of screening whereby women look and feel their breasts to detect any irregular developments including “lumps, distortions, discharges or swellings with an intention to detect it early for early initiation of treatment and better chance of survival for breast cancer patients” [4].

Even though BSE is a simple, quick, and cost-free procedure, its practice varies across different contexts. Previous studies conducted in Cameroon [12], Ghana [13], and Libya [14] have reported the prevalence of the practice of BSE to be 38.5%, 37.6%, and 12.1% respectively. These findings suggest that there are differences in the determinants of women’s practice of BSE. Nevertheless, after an extensive literature search, no study in Namibia has investigated the determinants of BSE among women. Evidence from neighbouring sub-Saharan African (SSA) countries has also focused mainly on awareness about BSE [12, 14]. The few studies from SSA that have investigated the phenomenon did not use nationally representative data or delimited the study to only students. Thus, reinforcing the need to unearth the determinants of BSE practices among women using a population-based dataset. The aim of this study is to examine the prevalence and determinants of BSE practices among women of reproductive age (15–49 years) in Namibia.

## Methods

### Data and materials

This study relied on the 2013 Namibia Demographic and Health Survey (NDHS). Specifically, data from the individual recode (IR) file was used. The Ministry of Health and Social Services (MoHSS) carried out the survey in collaboration with the Namibia Statistics Agency (NSA) and the National Institute of Pathology (NIP), with technical assistance provided by Inner City Fund (ICF) International and financial support provided by the Government of Namibia, the United States Agency for International Development (USAID), and the Global Fund [15]. A two-stage stratified sampling technique was used: stage 1 involved the selection of primary sampling units from urban (269) and rural areas (285) while stage 2 involved the selection of a fixed number of households (20) from both the urban and rural clusters. Details of the DHS methodology have been reported in previous studies [16, 17]. Data from 9,176 reproductive women aged 15–49 were included in this study. This study follows the ‘Strengthening the Reporting of Observational Studies in Epidemiology’ (STROBE) statement [18].

### Overview of Namibia

Namibia is a small country with a population of approximately 2.5 million people, bordering Zambia and Angola to the north, Botswana to the east, South Africa to the south and east, and the Atlantic Ocean to the west [15]. The country is divided into 14 administrative regions (Caprivi, Erongo, Hardap, Karas, Kavango West, Kavango East, Khomas, Kunene, Ohangwena, Omaheke, Omusati, Oshana, Oshikoto, and Otjozondjupa), with Windhoek, the capital city, located in the Khomas region. Windhoek, the country's largest city, is located in the central highlands. The main languages spoken are English, Ovambo, Khoekhoe, and Afrikaans [15].

### Measures

#### Outcome variable

Our outcome variable was BSE. This was assessed based on the question: “Have you ever examined own breasts for breast cancer?” The response options were “Yes” or “No”.

#### Explanatory variables

The explanatory variables included age, educational attainment, marital status, religion, parity, health insurance coverage, wealth index, mobility, contraceptive use, region of residence, early sexual debut, sex of household head, place of residence, listening to radio, watching TV, reading newspaper, having big problem with distance to healthcare facility, self-autonomy in healthcare decisions, and employment status [19–21]. These variables were measured in a binary (e.g., mobility: “Yes” or “No”) or multicategory (e.g., age: “15–19, 20–24, 25–29” etc.) format. Except for mobility, parity, and early sexual debut, the variables were used as they were originally coded according to their initial classification. Mobility was recoded from the variable asking participants about the total number of trips they had taken in the last 12 months. Participants who took no trip were classified under “No trip,” while those who took a single trip were classified under “one trip” and the last group who embarked on more than one trip were classified under “two or more.” Parity was created from the variable asking participants to provide the total number of children they ever gave birth to. With this variable, those who had never given birth were grouped as “none,” those who have had just one child as “one child,” those who have had two children as “two children,” those with three as “three children,” and those with more than three as “four and above.” Per the recommended definition of early sexual debut [22, 23], women who had no sexual intercourse experience were grouped under “no intercourse,” those who initiated their first sexual intercourse before the age of 15 were grouped under “less than 15 years,” those after the age of 15 and before 18 were grouped under “15–17 years” and lastly those after the age of 17 were group in the “18 and above” category. The covariates' classification is shown in Table 1.

**Table 1:** Weighted summary statistics of study variables with chi-square results (*N* = 9,176)

	Total n (%)	Breast Self-Examination	
		No (%)	Yes (%)
	9176 (100)	6262 (69.33; 95% CI = 67.82, 70.79)	2907 (30.67; 95% CI = 29.21, 32.18)
**Explanatory variables**			
**Age**		χ^2^ = 445.82, *p* ≤ 0.001	
15–19	1906 (20.77)	86.26	13.74
20–24	1786 (19.46)	73.73	26.27
25–29	1489 (16.23)	67.28	32.72
30–34	1260 (13.73)	62.77	37.23
35–39	1110 (12.1)	59.46	40.54
40–44	916 (10)	58.8	41.2
45–49	708 (7.72)	57.6	42.4
**Educational level**		χ^2^ = 311.91, *p* ≤ 0.001	
No education	419 (4.56)	82.72	17.28
Primary	1798 (19.6)	79.18	20.82
Secondary	6029 (65.71)	68.69	31.31
Higher	930 (10.13)	48.24	51.76
**Marital status**		χ^2^ = 251.24, *p* ≤ 0.001	
Never in union	5458 (59.48)	74.58	25.42
Married	1644 (17.92)	55.18	44.82
Living with partner	1476 (16.09)	69.08	30.92
Widowed	189 (2.06)	65.08	34.92
Divorced	93 (1.01)	51.37	48.63
Separated	315 (3.43)	60.95	30.05
**Religion**		χ^2^ = 75.46, *p* ≤ 0.001	
Roman Catholic	1802 (19.69)	75.37	24.63
Protestant	19.47 (21.27)	69.16	30.84
Elcin	4035 (44.09)	68.92	31.08
Seventh-day Adventist	436 (4.76)	70.61	29.39
No religion/Other	105 (1.15)	64.08	35.92
Other	827 (9.03)	58.88	41.12
**Health insurance coverage**		χ^2^ = 435.54, *p* ≤ 0.001	
No	7554 (82.39)	73.97	26.03
Yes	1614 (17.61)	47.57	52.43
**Employment**		χ^2^ = 311.42, *p* ≤ 0.001	
No	5202 (57.04)	76.67	23.33
Yes	3918 (42.96)	59.45	40.55
**Mobility in the last 12 months**		χ^2^ = 168.98, *p* ≤ 0.001	
No	5770 (62.93)	73.83	26.17
One trip	1507 (16.44)	65.83	34.17
Two trips and above	1892 (20.63)	58.44	41.56
**Early sexual debut**		χ^2^ = 275.13, *p* ≤ 0.001	
No Intercourse	1276 (14)	87.05	12.95
Less than 15 years	425 (4.66)	74.92	25.08
15–17	2844 (31.19)	70.43	29.57
18 years and above	4573 (50.15)	63.27	36.73
**Parity**		χ^2^ = 226.30, *p* ≤ 0.001	
None	2954 (32.19)	79.29	20.71
One birth	1871 (20.39)	65.38	34.62
Two births	1609 (17.53)	64.61	35.39
Three births	1140 (12.42)	59.15	40.85
Four births and above	1603 (17.47)	67.5	32.5
**Sex of household head**		χ^2^ = 11.78, *p* = *.01*	
Male Household	4122 (44.92)	67.49	32.51
Female Household	5054 (55.08)	70.82	29.18
**Contraceptive use**		χ^2^ = 103.92, *p* ≤ 0.001	
No Method	4572 (49.83)	74.25	25.75
Non-Modern Method	42 (.46)	60.32	39.68
Modern Method	4562 (49.72)	64.48	35.52
**Medical self-autonomy**		χ^2^ = 4.54, *p* = *0.16*	
Not a big problem	8595 (93.73)	69.06	30.94
Big Problem	574 (6.27)	73.29	26.71
**Distance to health facility**		χ^2^ = 92.93, *p* ≤ 0.001	
Not a big problem	6369 (69.45)	66.25	33.75
Big Problem	2802 30.55)	76.32	23.68
**Household wealth index**		χ^2^ = 528.09, *p* ≤ 0.001	
Poorest	1429 (15.57)	85.2	14.8
Poorer	1625 (17.71)	77.61	22.39
Middle	1795 (19.56)	72.4	27.6
Richer	2116 (23.06)	67.19	32.81
Richest	2211 (24.1)	69.33	47.5
**Reading of newspaper**		χ^2^ = 260.42, *p* = 0.001	
Not at all	2733 (29.82)	78.29	21.71
Less than once a week	2850 (31.1)	72.35	27.65
At least once a week	3581 (39.08)	60.04	39.96
**Listening to radio**		χ^2^ = 33.73, *p* ≤ 0.001	
Not at all	1601 (17.46)	73.76	26.24
Less than once a week	2273 (24.8)	71.6	28.4
At least once a week	5294 (57.75)	67	33
**Watching of television**		χ^2^ = 272, *p* ≤ 0.001	
Not at all	4123 (44.97)	77.46	22.54
Less than once a week	1214 (13.24)	69.96	30.04
At least once a week	3831 (41.79)	60.39	39.61
**Place of residence**		χ^2^ = 344, *p* ≤ 0.001	
Urban	5190 (56.56)	61.49	38.51
Rural	3986 (43.44)	79.52	20.48
**Region of residence**		χ^2^ = 728.84, *p* ≤ 0.001	
Caprivi	457 (5)	72.83	27.17
Erongo	771 (8.4)	48.04	51.96
Hardap	304 (3.31)	63.46	36.54
Karas	343 (3.74)	53.82	46.18
Kavango	836 (9.1)	89.59	10.41
Khomas	2202 (24)	58.2	41.8
Kunene	258 (2.81)	61.92	38.08
Ohangwena	894 (9.75)	85.08	14.92
Omaheke	225 (2.45)	55.23	44.77
Omusati	884 (9.63)	82.25	17.75
Oshana	755 (8.23)	76.22	23.78
Oshikoto	707 (7.7)	75.02	24.98
Otjozondjupa	540 (5.89)	69.01	30.99

*OR* Odds ratio, χ^2^ Chi-square value, *p* level of significance

### Statistical analyses

STATA Version 14 was used for data cleaning and analysis. Before performing the main data analysis, the data was cleaned, and variables were recoded. The primary data analyses employed bivariate, and multivariate analytical techniques. Prior to conducting these analyses, we used the complex survey mode command "svyset" to correct the NDHS's clusters, stratification, and sample weights. Because of the dataset's complex sampling design, this type of correction is recommended to control potential analytical errors and draw statistically sound conclusions. The next step was to run univariate analyses on the study's variables, generating weighted frequencies, percentages, and (where necessary) confidence intervals (lower and upper 95% confidence intervals). The Chi-square test of independence was then used to investigate the bivariate relationship between the variables in the study. Following that, a multivariate logistic regression analysis was performed to predict the outcome variable based on the explanatory and covariate variables. For the regression analysis, crude and adjusted odds ratios (OR) were reported.

### Ethical consideration

All methods were carried out in accordance with relevant guidelines. Ethical approval was not sought for this study since the data is freely available in the public domain. From the DHS reports, ethical clearance was sought and all ethical guidelines governing the use of human subjects in research were strictly adhered to. The detailed ethical guidelines are available at http://goo.gl/ny8T6X.

### Availability of data and materials

The data for this study is available on the DHS dataset website: http://dhsprogram.com/data/available-datasets.cfm.

## Results

### Sample description

Less than half (30.67%) of Namibian women of reproductive age have ever performed BSE. Approximately 18% of the participants have health insurance coverage. Most (20.77%) of the participants’ ages fall between 15 and 19 years. Many of the participants had obtained secondary school level education (65.71%), were unemployed (57.04%), were never married (59.48%), had not engaged in any trip for the past 12 months (62.93%), had their first sexual intercourse at 18 years or above (50.15%), followed the Elcin religion (44.09%), come from the wealthiest household (24.1%), lived in urban areas (56.56%) and were from the Khomas region (24%). The remaining results are shown in Table 1.

### Association between study variables

Age, education, marital status, health insurance, religion, employment, mobility, early sexual debut, parity, sex of household, contraceptive use, distance to health facility, wealth index, newspaper, radio and television exposure, and place and region of residence were all significantly associated with BSE. As seen in Table 1, a significant high proportion of the participants with health insurance coverage (26.03%), within 35 to 39 years (40.54%), with higher level of education (51.76%), who are employed (40.55%), who are divorced (48.63%), who engage in two trips and above in the last 12 months (41.56%), who sexually debuted at the age of 18 years and above (36.73%), have three children (40.85%), use non-modern contraceptives (39.68%), who belong to the richest households (47.5%), read newspaper at least once a week (39.96%), listen to radio at least once a week (33), watch television at least once a week (39.61%), reside in urban areas (38.51%) and reside in Erongo (51.96%) performed BSE.

### BSE in a bivariate and multivariate logistic model

A logistic regression model was used to investigate the correlates of BSE. Table 2 shows bivariate (odds ratio [OR] model) and multivariate (adjusted odds ratio [AOR] model) analyses for BSE. While the multivariate analysis produced the most interesting results, some significant associations in the bivariate analysis are worth noting. For example, age, education, health insurance coverage, mobility in the last 12 months, education, early sexual debut, parity, marital status, household wealth, newspaper, radio and television exposure, place and region of residence.

**Table 2:** Bivariate and multivariate logistic regression models of BSE amongst Namibia women

	Crude model			Adjusted model		
	***B***	**OR [95% CI]**	***p***	***B***	**AOR [95% CI]**	***p***
**Explanatory variables**						
**Age**						
15–19	1			1		
20–24	0.81	**2.23 [1.85, 2.71]**	** ≤ 0.001**	**0.31**	**1.35 [1.06, 1.72]**	**0.013**
25–29	1.12	**3.05 [2.49, 3.75]**	** ≤ 0.001**	**0.56**	**1.75 [1.33, 2.32]**	** ≤ 0.001**
30–34	1.31	**3.72 [3.05, 4.55]**	** ≤ 0.001**	**0.76**	**2.13 [1.57, 2.88]**	** ≤ 0.001**
35–39	1.45	**4.28 [3.44, 5.32]**	** ≤ 0.001**	**0.97**	**2.63 [1.88, 3.68]**	** ≤ 0.001**
40–44	1.48	**4.40 [3.51, 5.51]**	** ≤ 0.001**	**0.93**	**2.53 [1.74, 3.68]**	** ≤ 0.001**
45–49	1.53	**4.62 [3.66, 5.84]**	** ≤ 0.001**	**1.05**	**2.85 [1.97, 4.11]**	** ≤ 0.001**
**Educational level**						
No education	1			1		
Primary	0.23	1.26 [0.94, 1.69]	0.129	0.60	1.83 [1.34, 2.49]	** ≤ 0.001**
Secondary	**0.78**	**2.18 [1.65, 2.89]**	** ≤ 0.001**	**0.89**	**2.44 [1.78, 3.35]**	** ≤ 0.001**
Higher	**1.64**	**5.14 [3.59, 7.36]**	** ≤ 0.001**	**1.22**	**3.39 [2.24, 5.14]**	** ≤ 0.001**
**Marital Status**						
Never in union	1		1			
Married	**0.87**	**2.38 [2.03, 2.80]**	** ≤ 0.001**	0.72	1.07 [0.87, 1.32]	0.497
Living with partner	**0.27**	**1.31 [1.13, 1.53]**	** ≤ 0.001**	0.06	1.06 [0.89, 1.28]	0.501
Widowed/divorced/separated	**0.45**	**1.57 [1.12, 2.21]**	** ≤ 0.01**	0.13	1.14 [0.75, 1.75]	0.542
Divorced	**1.02**	**2.78 [1.61, 4.78]**	** ≤ 0.001**	0.12	1.13 [0.64, 2.02]	0.673
Separated	**0.63**	**1.88 [1.47, 2.40]**	** ≤ 0.001**	**0.31**	**1.36 [1.03, 1.80]**	**0.029**
**Religion**						
Roman Catholic	**-0.32**	**0.72 [0.62, 0.84]**	** ≤ 0.001**	**-0.18**	**0.84 [0.71, 0.98]**	**0.031**
Protestant	-0.1	0.99 [0.84, 1.16]	0.891	-0.05	0.95 [0.79, 1.13]	0.562
Elcin	1	1				
Seventh-day Adventist	-0.80	0.92 [0.67, 1.26]	0.617	-0.03	0.97 [0.69, 1.36]	0.861
No religion	0.22	1.24 [0.58, 2.65]	0.573	0.31	1.36 [0.68, 2.75]	0.384
Other	**0.44**	**1.55 [1.21, 1.98]**	** ≤ 0.001**	0.11	1.12 [0.89, 1.41]	0.332
**Health Insurance coverage**						
No	1			1		
Yes	**1.14**	**3.13 [2.67, 3.68]**	** ≤ 0.001**	**0.47**	**1.59 [1.34, 1.89]**	** ≤ 0.001**
**Employment**						
No	1			1		
Yes	**0.81**	**2.24 [1.99, 2.53]**	** ≤ 0.001**	0.03	1.02 [0.89, 1.19]	0.709
**Mobility in the last 12 months**						
No	**1**			**1**		
One trip	**0.38**	**1.46 [1.26, 1.70]**	** ≤ 0.001**	**0.20**	**1.22 [1.03, 1.43]**	**0.019**
Two trips and above	**0.70**	**2.01 [1.72, 2.33]**	** ≤ 0.001**	**0.22**	**1.25 [1.09, 1.43]**	** ≤ 0.001**
**Early sexual debut**						
No intercourse	**1**			**1**		
Less than 15	**0.81**	**2.25 [1.67, 3.03]**	** ≤ 0.001**	**0.54**	**1.71 [1.18, 2.47]**	** ≤ 0.01**
15–17	**1.04**	**2.82 [2.28, 3.48]**	** ≤ 0.001**	**0.49**	**1.64 [1.23, 2.18]**	** ≤ 0.001**
18 and above	**1.36**	**3.90 [3.17, 4.80]**	** ≤ 0.001**	**0.42**	**1.52 [1.13, 2.04]**	** ≤ 0.01**
**Parity**						
None	**1**			**1**		
One child	**0.71**	**2.03 [1.71, 2.40]**	** ≤ 0.001**	**0.42**	**1.51 [1.23, 1.87]**	** ≤ 0.001**
Two children	**0.74**	**2.10 [1.76, 2.50]**	** ≤ 0.001**	0.24	1.28 [0.99, 1.65]	0.063
Three children	**0.97**	**2.64 [2.18, 3.21]**	** ≤ 0.001**	**0.38**	**1.46 [1.08, 1.98]**	**0.013**
Four and above	**0.61**	**1.84 [1.55, 2.19]**	** ≤ 0.001**	**0.31**	**1.36 [1.02, 1.83]**	**0.039**
**Sex of the household head**						
Male Household	**1**			**1**		
Female Household	**-0.16**	**0.86 [0.76, 0.96]**	**0.011**	-0.01	1.00 [0.87, 1.14]	0.971
**Contraceptive use**						
No method	**1**			**1**		
Non-modern method	**0.64**	1.90 [0.93, 3.85]	0.076	0.39	1.48 [0.59, 3.66]	0.400
Modern method	**0.42**	**1.59 [1.42, 1.78]**	** ≤ 0.001**	0.02	1.02 [0.89, 1.17]	0.776
**Medical self-autonomy**						
Not a big problem	**1**			**1**		
Big Problem	-0.21	0.81 [0.61, 1.09]	0.160	0.07	1.08 [0.82, 1.41]	0.585
**Distance to health facility**						
Not a big problem	**1**			**1**		
Big Problem	**-0.50**	**0.61 [0.52, 0.70]**	** ≤ 0.001**	-0.03	0.97 [0.84, 1.12]	0.677
**Household wealth index**						
Poorest	1			1		
Poorer	**0.51**	**1.66 [1.33, 2.07]**	** ≤ 0.001**	0.15	1.16 [0.91, 1.47]	0.224
Middle	**0.79**	**2.20 [1.75, 2.76]**	** ≤ 0.001**	0.22	1.24 [0.96, 1.61]	0.098
Richer	**1.03**	**2.81 [2.23, 3.54]**	** ≤ 0.001**	0.19	1.21 [0.91, 1.60]	0.182
Richest	**1.65**	**5.21 [4.16, 6.52]**	** ≤ 0.001**	**0.53**	**1.69 [1.23, 2.33]**	** ≤ 0.001**
**Reading of newspaper**						
Not at all	1			1		
Less than once a week	**0.32**	**1.38 [1.19, 1.60]**	** ≤ 0.001**	0.03	1.03 [0.87, 1.22]	0.691
At least once a week	**0.88**	**2.40 [2.04, 2.82]**	** ≤ 0.001**	0.13	1.14 [0.93, 1.39]	0.207
**Listening to radio**						
Not at all	1			1		
Less than once a week	0.11	1.12 [0.91, 1.36]	0.286	0.08	1.08 [0.85, 1.37]	0.509
At least once a week	**0.33**	**1.38 [1.17, 1.63]**	** ≤ 0.001**	-0.07	0.93 [0.77, 1.12]	0.459
**Watching of television**						
Not at all	1			1		
Less than once a week	**0.39**	**1.48 [1.24, 1.76]**	** ≤ 0.001**	-0.11	0.90 [0.74, 1.09]	0.274
At least once a week	**0.81**	**2.25 [1.96, 2.59]**	** ≤ 0.001**	-0.02	0.98 [0.82, 1.19]	0.838
**Place of residence**						
Urban	**1**			1		
Rural	-0.89	**0.41 [0.36, 0.47]**	** ≤ 0.001**	-0.07	0.93 [0.77, 1.13]	0.465
**Region of residence**						
Caprivi	**-0.66**	**0.52 [0.36, 0.74]**	** ≤ 0.001**	-0.27	0.76 [0.51, 1.15]	0.193
Erongo	**0.41**	**1.51 [1.11, 2.05]**	** ≤ 0.01**	**0.50**	**1.66 [1.18, 2.32]**	** ≤ 0.01**
Hardap	-0.22	0.80 [0.63, 1.01]	0.064	-0.04	0.96 [0.77, 1.20]	0.727
Karas	0.18	1.19 [0.93, 1.53]	0.162	**0.36**	**1.43 [1.13, 1.82]**	** ≤ 0.01**
Kavango	**-1.82**	**0.16 [0.12, 0.22]**	** ≤ 0.001**	**-1.29**	**0.28 [0.21, 0.37]**	** ≤ 0.001**
Khomas	1			1		
Kunene	-0.16	0.86 [0.60, 1.23]	0.396	0.27	1.31 [0.86, 2.0]	0.204
Ohangwena	**-1.41**	**0.24 [0.17, 0.35]**	** ≤ 0.001**	**-0.82**	**0.44 [0.31, 0.63]**	** ≤ 0.001**
Omaheke	0.12	1.13 [0.83, 1.54]	0.446	**0.57**	**1.77 [1.29, 2.42]**	** ≤ 0.001**
Omusati	**-1.20**	**0.3 [0.22, 0.41]**	** ≤ 0.001**	**-0.67**	**0.51 [0.36, 0.73]**	** ≤ 0.001**
Oshana	**-0.83**	**0.43 [0.33, 0.56]**	** ≤ 0.001**	**-0.54**	**0.58 [0.46, 0.74]**	** ≤ 0.001**
Oshikoto	**-0.77**	**0.46 [0.35, 0.61]**	** ≤ 0.001**	**-0.36**	**0.70 [0.61, 1.04]**	** ≤ 0.01**
Otjozondjupa	**-0.47**	**0.63 [0.48, 0.82]**	** ≤ 0.001**	-0.23	0.79 [0.61, 0.07]	0.091

*OR* Odds ratio, *AOR* Adjusted odds ratio, *p* level of significance

In the multivariate analysis model, age, educational level, health insurance coverage, employment status, mobility in the last 12 months, early sexual debut, religion, parity, marital status, household wealth index and region of residence were significantly associated with BSE. Age was found to be significantly associated with BSE practice. From the findings, women with the age group of 20–49 years had a higher likelihood of performing BSE compared with those within the age group of 15–19 years. Those with secondary [AOR = 2.44, 95% CI:1.78, 3.35, *p* ≤ 0.001] or higher education [AOR = 3.39, 95%CI:2.24, 5.14, *p* ≤ 0.001] had higher odds of performing BSE than those with no educational background. Compared to women of reproductive age who live in Khomas, those living in Erongo [AOR = 1.66, 95% CI: 1.18, 2.32, *p* ≤ 0.01], Karas [AOR = 1.43, 95% CI: 1.13, 1.82, *p* ≤ 0.01], and Omaheke [AOR = 1.77, 95% CI: 1.29, 2.42, *p* ≤ 0.001], were more likely to practice BSE. On the other hand, those in Kavango [AOR = 0.28, 95% CI: 0.21, 0.37, *p* ≤ 0.001], Ohangwena [AOR = 0.44, 95% CI: 0.31, 0.63, *p* ≤ 0.001], Omusati [AOR = 0.51, 95% CI: 0.36, 0.73, *p *≤ 0.001], Oshana [AOR = 0.58, 95% CI: 0.46, 0.74, *p* ≤ 0.001], and Oshikoto [AOR = 0.70, 95% CI: 0.61, 1.04, *p* ≤ 0.01] were less likely to practice BSE compared to those in Khomas.

Also, women of reproductive age with health insurance coverage were found to have increased odds of performing BSE than women without health insurance coverage [AOR = 1.59, 95% CI: 1.34, 1.89, *p* ≤ 0.001]. Those who had taken one [AOR = 1.22, 95% CI: 1.03, 1.43, *p* ≤ 0.05] or more trips [AOR = 1.25, 95% CI: 1.09, 1.43, p ≤ 0.001] in the previous year had a higher likelihood of performing BSE than those who had not taken any trips. Women of reproductive age who had separated from their spouses were more likely than non-married women to perform BSE [AOR = 1.36, 95% CI: 1.03, 1.80, p ≤ 0.05]. Those who had their first sexual intercourse when they were less than 15 years [AOR = 1.64, 95% CI: 1.23, 2.18, *p* ≤ 0.001] to 18 years and above [AOR = 1.59, 95% CI: 1.13, 2.04, *p* ≤ 0.01] had higher odds of performing BSE than women of reproductive age who had no sexual intercourse. Compared to those from the poorest households, women of reproductive age in the richest households were more likely to perform BSE [AOR = 1.69, 95% CI: 1.23, 2.33, *p* ≤ 0.001]. Roman Catholic women were also less likely to perform BSE than those in the Elcin religion [AOR = 0.84, 95% CI: 0.71, 0.98, *p* ≤ 0.05]. Those with three [AOR = 1.46, 95% CI: 1.08, 1.98, *p* ≤ 0.05] or more children [AOR = 1.36, 95% CI: 1.02, 1.83, *p* ≤ 0.05] had a higher likelihood of performing BSE compared with those without children.

## Discussion

In resource-constrained settings like Namibia, BSE is considered an important cost-effective intervention that is critical to the early detection of breast cancer [24, 25]. However, this important public health issue has received little scholarly attention in Namibia. Hence, the present study sought to examine the prevalence and determinants of BSE practices among women of reproductive age (15–49 years) in Namibia. Our findings showed that less than half (30.67%) of Namibian women aged 15–49 years practiced BSE. A similar prevalence of BSE practice has been reported in Cameroon (38.5%) [26], and Ghana (37.6%) [13]. However, the estimated prevalence from this study is significantly lower when compared to the prevalence of BSE in Ethiopia (45.8%) [4]. This low prevalence of BSE among Namibian women of reproductive age is a threat to the early detection of breast cancer. If this situation is left unabated, then most women who develop the disease may be at high risk of metastatic breast cancer and have a highly negative prognosis. Probably, the low prevalence of BSE could be a reflection of low knowledge of the practice, or how it should be carried out [27].

Our study shows that age was a significant factor that predicted the practice of BSE among women of reproductive age in Namibia. Compared to adolescent girls, adult women of reproductive age (20–49 years) were more likely to perform BSE. For example, those aged between 45 and 49 years were 2.85 times more likely to practice BSE compared to adolescent girls (15–19 years). This finding is inconsistent with previous studies that have shown that as the age of a woman increases, their likelihood to practice BSE reduces significantly [13, 28]. We postulate that this counter-intuitive finding about the association between age and the BSE practice could be due to methodological differences. For instance, in the study conducted in Ghana, the age grouping began from < 30 years [13]. This means that adolescent girls and young women have been lumped together. Therefore, the true effect of the association between age and BSE practice may be masked. Nevertheless, our result that increasing age is associated with  the higher practice of BSE mirrors the findings of a study conducted among Jordanian women [29]. The findings may be explained from the perspective that the risk of developing breast cancer increases as one ages [30, 31]. Therefore, as women age, they become conscious about their risk of developing the disease and would therefore be more likely to perform BSE to facilitate early detection of any anomalies in the breast.

The study revealed that having formal education was significantly associated with higher odds of performing BSE, compared to those who had no formal education. This finding aligns with previous studies from Nigeria [32], Ethiopia [33], and Ghana [34] that have found the odds of practicing BSE to be significantly less among women who have no formal education. A plausible explanation for this could be that, women who have formal education have a better appreciation and understanding of health awareness information regarding the benefits of and processes involved in performing BSE [33]. Compared to women of reproductive age with no formal education, those who have gained some formal education have access to different sources of health information including television, Facebook, WhatsApp, and YouTube [13]. This has the tendency to increase the perceived self-efficacy of these women to perform BSE, hence, the higher odds of BSE practice.

It is evident from our findings that household wealth index is a significant predictor of BSE among Namibian women of reproductive age. Women aged 15–49 in the higher wealth index had higher odds of practicing BSE as compared to their counterparts who were in the poorest wealth index. Similar findings have been reported in Jordan [29] where women in the richest wealth index were 1.22 times more likely to practice BSE compared to those in the poorest wealth index. Relatedly, we found that women of reproductive age who were covered by health insurance were more likely to practice BSE. This is corroborated by previous studies that have indicated that health insurance coverage was significantly associated with the likelihood of women of reproductive age to practice BSE [19, 35]. A plausible explanation for this result could be that being in the higher wealth index offers women of reproductive age the opportunity to easily visit the health facility where they may be exposed to health information about BSE, how it should be done, and the benefits of practicing BSE. Similarly, health insurance serves as a pro-poor intervention that enables poor women of reproductive age to have access to health information that hitherto, would have been difficult for them to access [19].

We also observed a significant association between early sexual debut, parity and BSE practice. Women of reproductive age who initiated sex before age 15 had higher odds of performing BSE compared to those who had never had sexual intercourse. Perhaps this might be due to the low-risk perception of those who have had no sexual intercourse [36]. Also, compared to nulliparous women of reproductive age, those who had children were more likely to practice BSE. However, within the dimension of parity, we observed that the odds of performing BSE significantly reduced for multiparous women of reproductive age. That is, the higher the parity, the less likely women of reproductive age were to perform BSE. A related study conducted among women in rural Nigeria found similar findings that multiparous women were less likely to practice BSE as compared to uniparous women [37]. This could be explained from the perspective that multiparous women tend to perceive that they have had sufficient experience and information relating to their reproductive health as a result of the number of childbirths [38], hence, there is no need for them to practice BSE.

Our study further reveals that the region of residence predicted women’s practice of BSE. While women of reproductive age in Omaheke, Karas, and Erongo had significantly higher odds of practicing BSE, those residing in Kavango, Ohangwena, Omusati, Oshana, and Oshikoto had significantly lower odds of performing BSE. Perhaps, we may explain this result from the perspective that there are existent geographical disparities in health information across the regions of Namibia [39].

### Practical implication

Our findings underscore a need for the Namibian government and health department to invest in interventions to enhance women of reproductive age’s uptake of BSE. Interventions developed to facilitate BSE practice among Namibian women of reproductive age must be tailored to improve the economic situation of women. Practically, it could be achieved by empowering women of reproductive age with livelihood skills. Also, there is a need to prioritise formal and higher education for adolescent girls and young women as education emerged as a significant determinant of BSE practice. Advocacy and educational campaigns need to be directed at multiparous women of reproductive age and adolescent girls. The findings also highlight the role of health insurance coverage in promoting BSE practice. Therefore, the Namibian government must strengthen the health insurance system in the country. This could be achieved by implementing a national health insurance scheme as it is in countries like Ghana to bridge the existing inequalities in the current health insurance regime in Namibia.

### Strengths and limitations

The use of a nationally representative dataset supports the generalisability of the study findings. We performed appropriate statistical analyses which guarantee the validity and reliability of our findings. Notwithstanding, there are some limitations. The cross-sectional nature of the DHS does not allow us to establish causal inferences. Also, the analysis was restricted to only women who were in the reproductive age group (15–49 years). Therefore, our findings may not necessarily reflect the dynamics in the population that falls outside the scope of the reproductive age (15–49 years). We also acknowledge that there is the possibility of social desirability and recall bias that could have affected the results. Although we used the most current nationally representative dataset from Namibia, we do acknowledge that the time between 2013 and now might have brought about significant changes. Therefore, the findings about the geographical differences may have changed. Hence, interpretations must be made with caution.

## Conclusion

We conclude that the determinants of BSE practice are age, educational level, marital status, health insurance coverage, religion, mobility in the last 12 months, early sexual debut, parity, household wealth index, and region of residence. Any policy or intervention to improve BSE practice among Namibian women must target adolescent girls, women with no formal education, those without health insurance coverage, multiparous women, those in the poorest wealth index and those residing in Kavango, Ohangwena, Omusati, Oshana, and Oshikoto regions.

## Data Availability

Data for this study is available on the DHS dataset website: http://dhsprogram.com/data/available-datasets.cfm.

## Abbreviations

BSE: Breast Self-Examination
DHS: Demographic and Health Survey
ICF: Inner City Fund
LMICs: Low-and-middle-income countries
MoHSS: Ministry of Health and Social Services
NNCR: Namibian National Cancer Registry
NSA: Namibia Statistics Agency
NIP: National Institute of Pathology
USAID: United States Agency for International Development
STROBE: Strengthening the Reporting of Observational Studies in Epidemiology
